# Transcriptomic response of prostate cancer cells to carbon ion and photon irradiation with focus on androgen receptor and TP53 signaling

**DOI:** 10.1186/s13014-024-02480-z

**Published:** 2024-07-02

**Authors:** Jörg Hänze, Lilly M. Mengen, Marco Mernberger, Dinesh Kumar Tiwari, Thomas Plagge, Andrea Nist, Florentine S. B. Subtil, Ulrike Theiss, Fabian Eberle, Katrin Roth, Matthias Lauth, Rainer Hofmann, Rita Engenhart-Cabillic, Thorsten Stiewe, Axel Hegele

**Affiliations:** 1 Department of Urology, Faculty of Medicine, Philipps University Marburg, Baldingerstraße, 35043 Marburg, Germany; 2 Institute of Molecular Oncology, Genomics Core Facility, Member of the German Center for Lung Research (DZL), Philipps University Marburg, Marburg, Germany; 3 Department of Radiotherapy and Radiooncology, Philipps University Marburg, Marburg, Germany; 4 Marburg Ion-Beam Therapy Center (MIT), Department of Radiotherapy and Radiation Oncology, Marburg University Hospital, Marburg, Germany; 5 Core Facility Cellular Imaging, Philipps University Marburg, Marburg, Germany; 6 Center for Tumor and Immune Biology, Philipps University Marburg, Marburg, Germany; 7 Urological Center Mittelhessen, DRK Hospital Biedenkopf, Biedenkopf, Germany

**Keywords:** Carbon ion irradiation, Photon irradiation, Prostate cancer, Androgen receptor, TP53

## Abstract

**Background:**

Radiotherapy is essential in the treatment of prostate cancer. An alternative to conventional photon radiotherapy is the application of carbon ions, which provide a superior intratumoral dose distribution and less induced damage to adjacent healthy tissue. A common characteristic of prostate cancer cells is their dependence on androgens which is exploited therapeutically by androgen deprivation therapy in the advanced prostate cancer stage. Here, we aimed to analyze the transcriptomic response of prostate cancer cells to irradiation by photons in comparison to carbon ions, focusing on DNA damage, DNA repair and androgen receptor signaling.

**Methods:**

Prostate cancer cell lines LNCaP (functional TP53 and androgen receptor signaling) and DU145 (dysfunctional TP53 and androgen receptor signaling) were irradiated by photons or carbon ions and the subsequent DNA damage was assessed by immuno-cytofluorescence. Furthermore, the cells were treated with an androgen-receptor agonist. The effects of irradiation and androgen treatment on the gene regulation and the transcriptome were investigated by RT-qPCR and RNA sequencing, followed by bioinformatic analysis.

**Results:**

Following photon or carbon ion irradiation, both LNCaP and DU145 cells showed a dose-dependent amount of visible DNA damage that decreased over time, indicating occurring DNA repair. In terms of gene regulation, mRNAs involved in the TP53-dependent DNA damage response were significantly upregulated by photons and carbon ions in LNCaP but not in DU145 cells, which generally showed low levels of gene regulation after irradiation. Both LNCaP and DU145 cells responded to photons and carbon ions by downregulation of genes involved in DNA repair and cell cycle, partially resembling the transcriptome response to the applied androgen receptor agonist. Neither photons nor carbon ions significantly affected canonical androgen receptor-dependent gene regulation. Furthermore, certain genes that were specifically regulated by either photon or carbon ion irradiation were identified.

**Conclusion:**

Photon and carbon ion irradiation showed a significant congruence in terms of induced signaling pathways and transcriptomic responses. These responses were strongly impacted by the TP53 status. Nevertheless, irradiation mode-dependent distinct gene regulations with undefined implication for radiotherapy outcome were revealed. Androgen receptor signaling and irradiations shared regulation of certain genes with respect to DNA-repair and cell-cycle.

**Supplementary Information:**

The online version contains supplementary material available at 10.1186/s13014-024-02480-z.

## Introduction

Photon irradiation is an established technique for the treatment of various cancers including prostate cancer [1]. Carbon ion (^12^C-ion) irradiation by a particle accelerator is an innovative radiation technique in medicine with different physical properties allowing high precision targeting of the tumor region [2–4]. Particularly, the radiation energy of ^12^C-ions is directed as a Bragg-Peak to constrained predetermined tissue depths as opposed to the deposition of photon energy at broader tissue depths [5]. Thereby, ^12^C-ion irradiation can more precisely target the tumor volume while minimizing hazardous effects on healthy adjacent tissue. This may be superior for the irradiation of anatomically critical tumor entities such as those of the central nervous system, head-neck and prostate cancer [3, 6, 7].

Beside radiation physics, the genotoxic insults exerted by photon versus ^12^C-ion irradiation may have common and divergent characteristics in prostate cancer with impact on cellular responses of DNA-damage, DNA repair and gene regulation [8–10]. In addition, the responses may depend on the cancer cell specific context due to differences in the occurring genetic alterations as well as active signaling pathways that specifically drive tumor progression [11–13] potentially affecting the therapeutic outcome of irradiation.

In prostate cancer, irradiation therapy is a treatment option in all clinical stages of the disease [1]. One significant aspect of prostate cancer is the androgen receptor (AR) signaling pathway which is exploited as a drug target by pharmacologic androgen deprivation or antagonistic AR blockade therapy in certain tumor stages [14–16]. Critically, AR signaling has been shown to be interconnected with DNA-damage and with DNA-repair genes in prostate cancer cells and tumor models. In primary human prostate cancer samples, a link between AR signature and DNA repair genes was demonstrated. Combined AR intervention and irradiation resulted in decreased clonogenic survival of prostate cancer cells or tumor progression in mice models. Notably, the protocols of AR intervention included both antagonistic inhibition or agonistic activation of AR signaling in different doses [8–10]. Overall, these findings corroborate that intervention in AR signaling can favor the outcome of photon irradiation therapy. However, it remains unknown whether the gene regulatory effects are transferable to irradiation using ^12^C-ions.

Of further relevance is the prominent tumor suppressor TP53 pathway, which functions as a critical master of gene regulation [17–19]. In general, TP53 is crucial for therapeutic cancer interventions that target DNA integrity such as chemotherapeutics and irradiation. TP53 is activated by DNA damage such as DNA double strand breaks (DSB) and induces cell cycle arrest through a well-defined signaling cascade. TP53 is regulated by a feedback circuit involving MDM2 ubiquitination and proteasomal degradation. The cell cycle kinase inhibitor (CDKN1A) is a key target gene of TP53 that initiates the cell cycle arrest.

In prostate cancer, TP53 is dysfunctional in a considerable subset of cases in addition to AR mutations. Both TP53 and AR aberrations indicate a worse prognosis in metastatic androgen responsive prostate cancer [20].

Here, we aimed to explore common and selective effects of ^12^C-ion and photon irradiation with respect to DNA-damage and gene regulation, as reflected by alterations of mRNA levels in two human prostate cancer cell lines. We analyzed LNCaP cells with functional TP53 and AR, as well as DU145 cells with dysfunctional TP53 and AR. In particular, we focused on target genes related to DNA repair, cell cycle and DNA replication. Alongside, we monitored AR signaling that was induced as reference by dihydrotestosterone in LNCaP cells and examined correlations with the respective irradiation responses. These analyses were complemented by whole transcriptome analyses using RNA-seq technique. In essence, we identified common and unique changes in gene regulation induced by the different irradiation modes.

## Materials and methods

### Culturing of cell lines

The human prostate cancer cell lines LNCaP and DU145 were obtained and authenticated from the *Leibniz Institute DSMZ-German Collection of Microorganisms and Cell Cultures, Germany* (LNCaP, DSMZ-ACC 256; DU145, DSMZ-ACC 261). The shipment of LNCaP cells was in September 2017 and the latest authentication of DU145 cells in November 2018. Authentication was performed by DNA profiling using different and highly polymorphic short tandem repeat (STR) loci. The cell culture experiments were performed between 2018 and 2020. The experiments were performed with tested mycoplasma-free cells (Minerva Biolabs GmbH, Berlin, Germany). LNCaP cells have functional androgen receptor and TP53 signaling. DU145 cells are non-responsive to androgens and are non-functional for TP53 due to a missense mutation (cBioPortal v4.0.4: Cancer Cell Line Encyclopedia [21]). Cells were cultured in accordance with the recommended conditions in RPMI full growth medium supplemented with fetal bovine serum. LNCap and DU145 cells were treated by irradiations and LNCaP cells by addition of (5α, 17β)-17-hydroxy-androstane-3-one (di-hydro-testosterone; DHT) (20 nM) (Sigma-Aldrich). All treatments were performed in three independent triplicates with a calculated cell density of approximately 50% confluence at the time point of setup.

### RNA and protein isolation

RNA was isolated by the RNeasy Mini Kit (QIAGEN, Hilden, Germany) procedure according to the manufacturer’s protocol. Protein was isolated with RIPA buffer (Cell signalling Technology Europe, Frankfurt a.M., Germany) supplemented with protease inhibitor cocktail (Sigma-Aldrich Chemie, GmbH, Munich, Germany). The isolation procedure was performed according to the manufacturer’s protocols.

### Western blot

Protein samples (40 µg) were analysed by sodium dodecyl sulphate polyacrylamide gel electrophoresis with subsequent electric transfer to a nitrocellulose membrane (Bio-Rad-System, Germany). The membranes were blocked in TRIS-buffered saline with 0.1% tween containing 5% dry milk and the primary antibodies were added and incubated at 4 °C for 24 h. The antibodies were as follows: H2A.X, rabbit mAb, #7631S, dilution 1:5000, Cell Signaling Technology; Phospho-Histone H2A.X (Ser139), rabbit mAb, #9718S, dilution 1:5000, Cell Signaling Technology; cytoplasmic β-actin, mouse #MAK6019 Linaris GmbH. Then, the respective secondary anti host antibodies coupled with horseradish peroxidase (Anti-Rabbit IgG: HRP, goat, #ZRH1158, Linaris; anti-Mouse IgG: HRP, goat, #31,430, dilution 1:5000, Thermo Scientific) were added for band detection with enhanced chemiluminescent luciferase kit (Thermo Scientific, Rockford, USA) by an imager system (Fluorchem IS-8900, Alpha Innotech, San Leandro, CA, USA).

### cDNA synthesis and realtime RT-PCR

RNA (1 µg) was treated with DNAse I. cDNA synthesis was performed with random hexamer primers and M-MLV reverse transcriptase. The cDNA was submitted to SYBR green (ThermoScientific, UK) based RT-qPCR (IQ5, Biorad, Germany). Cycling conditions were: 95 °C, 7.5 m followed by 40 cycles (95 °C, 15 s; 58 °C, 30 s; 72 °C, 30 s). Melting curve analysis was performed by a temperature increment (0.5 °C, 10 s) from 60 up to 95 °C. Target mRNA levels are displayed as -ΔCt values (log 2-scale) normalized to TATA-binding protein (TBP)-mRNA as references. The primer sets (Biomers GmbH, Germany) were derived from sequence entries in GenBank and selected by Primer-Blast (NCBI National Center for Biotechnology Information). The corresponding sequences are listed (supplementary Table S). The RT-PCR amplicons were characterized by DNA length and melting temperature profile and contaminations were excluded by appropriate controls (data not shown).

### Irradiations

For photon irradiation an X-RAD 320ix cabinet was used (X-ray unit (Precision X-Ray Inc, Denver, USA, 320 kV, 8 mA, filter: 0.5 mm Cu and 0.5 mm Al, dose rate 1 Gy/min).

^12^C-irradiation was performed at the Marburg Ion-Beam Therapy Centre (MIT). Cells were irradiated with a horizontal beam of 114.5–129.5 MeV/n ^12^C-ions and positioned in the middle of a spread-out Bragg peak (SOBP) of 10–20 mm. Fields were applied using active scanning with a square of 324 cm^2^.

### Immuno-cytofluorescence of γ-H2AX/53BP1 DSB foci

For detection of DSB repair foci, co-staining with *γ*-H2AX and 53BP1 antibodies was performed. Asynchronous growing cells were seeded on glass cover slips 36 h prior to irradiation [22–24]. The cover slips were precoated by 0.01% Poly-L-Lysin-hydrobromide for improving cell adherence to the surface. Cells were fixed and stained at various time points (2–48 h) after irradiation using 4% para-formaldehyde/PBS for 10 min. Fixed cells were permeabilized with 0.2% Triton X-100, 1% BSA/PBS for 10 min, washed with 1% BSA/PBS and blocked in 3% BSA/PBS for 1 h. The primary antibody solution was incubated for 1.5 h at room temperature using the following antibodies: mouse monoclonal anti-phospho-S139-H2AX antibody (1:500, clone JBW301, Millipore, Darmstadt, Germany) and rabbit polyclonal 53BP1 antibody (1:500, Novus Biologicals, Wiesbaden, Germany). After washing three times with 0.1% Tween20/PBS for 10 min, the cells were incubated for 1.5 h with secondary anti- mouse Alexa-fluor594 (1:1000) and anti-rabbit Alexa-fluor488 (1:1000, both Invitrogen, Karlsruhe, Germany). Cells were again washed three times and mounted in ProLong Gold antifade reagent (Invitrogen, Karlsruhe, Germany) containing DAPI for staining of nuclei. Immunofluorescence analysis was conducted using the Leica DM5500 wide-field microscope. About twenty z-stacks (0.30 mm) per image were captured using an immersion objective with 63 × magnification and a numerical aperture of 1.25. To analyse the z-stacked images, they were deconvoluted and overlaid with the integrated LAS-AF software (Leica, Wetzlar, Germany), and foci were counted using FiJi software.

### Statistical analysis

Correlation analyses was performed by non-parametric Spearman test and linear regression (Pearson correlation) analysis. The analyses were performed with MS Excel 2017 and Graphpad Prism 9.5.1.

### RNAseq

For RNAseq, RNA quality was assessed using the Bioanalyzer RNA 6000 Nano Kit (Agilent). RNAseq libraries were prepared from total RNA with the QuantSeq 3′ mRNA-Seq Library Prep Kit FWD for Illumina (Lexogen) in combination with the UMI Second Strand Synthesis Module for QuantSeq FWD (Illumina, Read 1) (Lexogen) according to the manufacturer’s instructions. Quality of sequencing libraries was controlled on a Bioanalyzer 2100 using the Agilent High Sensitivity DNA Kit (Agilent). Pooled sequencing libraries were quantified and sequenced on the NextSeq550 platform (Illumina) with 75 bases single reads. The raw data has been submitted to EMBL Biostudies and can be accessed via the accession number E-MTAB-14099.

### Data analyses and bioinformatics of RNA seq data

Unique molecular identifiers (UMI) were extracted from the sequenced reads and the first four nucleotides corresponding to the QuantSeq FWD-UMI 3′ spacer were removed. Trimmed reads were mapped to the Homo sapiens (revision 99, GRCh38) Ensembl reference genome, using STAR (version 2.6.1d) [25]. After alignment, UMIs were deduplicated using UMI-tools (version 1.1.1). UMI per gene were quantified and normalized to counts per million (CPM). Genes with CPM counts were below 1 in all samples were considered background noise and discarded. In addition, genes were restricted to protein-coding genes in the further analysis. Raw read counts of paired samples were used to assess differential gene expression via EdgeR (version 3.24.0). Obtained p-values were corrected via Benjamini–Hochberg correction. Genes with log2FC ≥ 1 as well as corrected *p*-values < 0.05 were considered differentially expressed. Principle component analysis was performed using scikit-learn. Gene set enrichment analysis [26] was performed using gene sets from Molecular Signatures Database (MSigDB: https://www.gsea-msigdb.org/gsea/msigdb/ index.jsp) and from [9] as listed. The conclusive parameters of GSEA comprise the enrichment score (ES) that can be positive or negative, the false discovery rate (FDR q-value) and the nominal *p*-value. ES-values with FDR < 0.25 and nominal *p*-value < 0.05 are considered as significant. The ES values are displayed in volcano-plots (ES vs − log FDR) (Fig. 4). For GSEA-graphs and parameters see supplementary information.

## Results

**Fig. 1:**
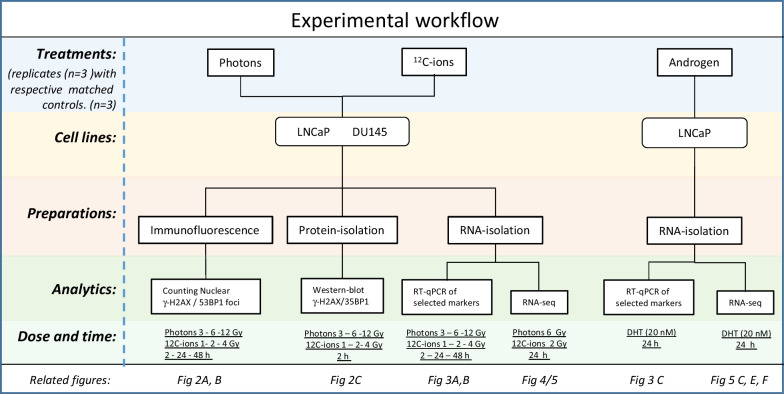
Schematic diagram of the experimental workflow

We analyzed cellular and molecular effects triggered by irradiation with photons or ^12^C-ions. We focused on DNA damage as well as selective and comprehensive gene regulation. For that, we analyzed the two cell lines LNCaP and DU145 that differ genetically with respect to TP53 and androgen signaling. LNCaP have functional TP53 and androgen receptor (AR) signaling whereas DU145 are dysfunctional for TP53 and AR signaling. AR signaling was induced in LNCaP cells by addition of dihydrotestosterone (DHT). A schematic diagram summarizes the experimental workflow (Fig. 1).

### DNA damage evaluated by immuno-cytofluorescence of γ-H2AX and 53BP1

**Fig. 2:**
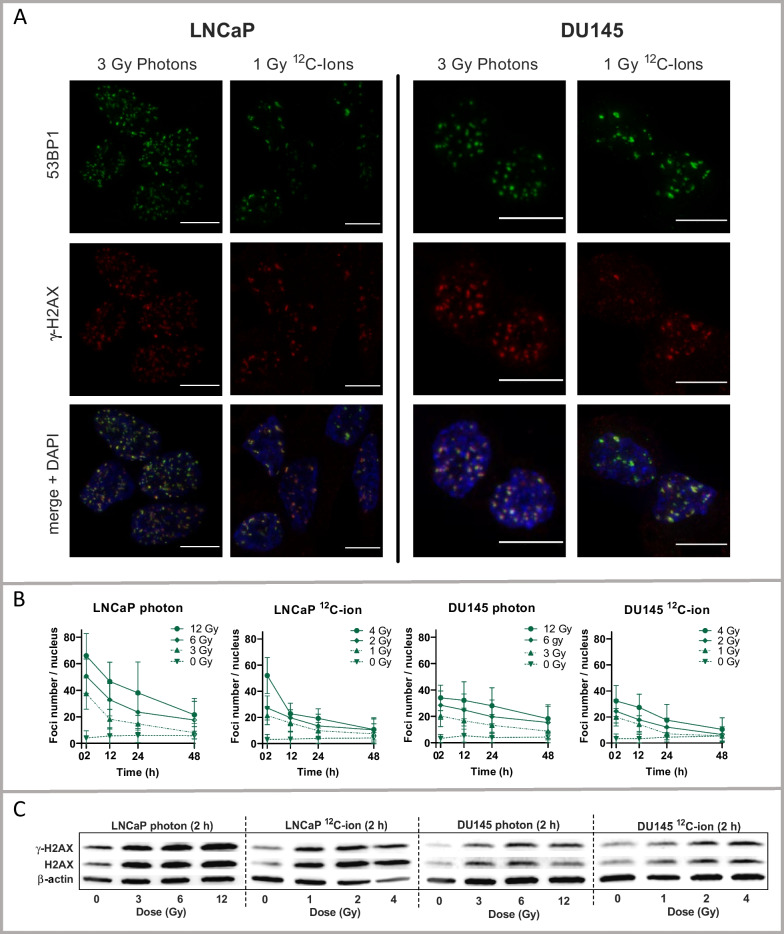
Analysis of DNA-damage in LNCaP and DU145 cells following photon and ^12^C-ion irradiation. **A** Immunofluorescence of *γ-*H2AX (red), 53BP1 staining (green) and DAPI-merge (weakly blue) for counterstaining of nuclei. The co-localized red and green foci indicate the DNA-double-strand-breaks (DSB) per nucleus. The white bar scale corresponds to 10 µm. The respective images of dose and time served for counting of DSB-foci. **B** Number of DSB-foci for photon and ^12^C-ion irradiation of LNCaP (left) and DU145 (right) time dependently for each dose of photon or ^12^C-ion irradiation (mean ± SD, n ≥ 95). **C** Western-blot analysis of *γ-*H2AX and H2AX, after (2 h) photon or ^12^C-ion irradiation, dose-dependent in LNCaP cells (left) and DU145 cells (right)

We measured DNA-damage caused by ^12^C-ion or photon irradiation by immunofluorescence for *γ-H2AX and 53BP1* indicating the DNA-double-strand breaks (DSB) (Fig. 2A). The applied dose ranges for ^12^C-ions (0, 1, 2, 4 Gy) and photons (0, 3, 6, 12 Gy) were adjusted to take account of the relative biological effectiveness (RBE), which is higher for ^12^C-ions (approximately 3-times) than for photons [23, 27]. Using the number of DSB-foci as an indicator for DNA damage, the respective ^12^C-ion and photon dose curves strikingly overlapped for LNCaP and DU145 cells (Fig. 2B). Typically, the number of DSB-foci at 2 h after irradiation correlated with the irradiation dose. The numbers of DSB-foci per nucleus were higher in LNCaP than in DU145 cells. The decrease in DSB-foci over time reflected the process of DNA repair. Alongside, the formation of DSB-foci was paralleled by an increase of protein abundance of *γ-*H2AX and H2AX in cell lysates of LNCaP and DU145 cells (Fig. 2C) upon photons and ^12^C-ions.

### *Regulation of mRNAs encoding genes for DNA damage, DNA repair, DNA replication and cell cycle after photon and *^*12*^*C-ion irradiation in LNCaP and DU145 cells*

**Fig. 3:**
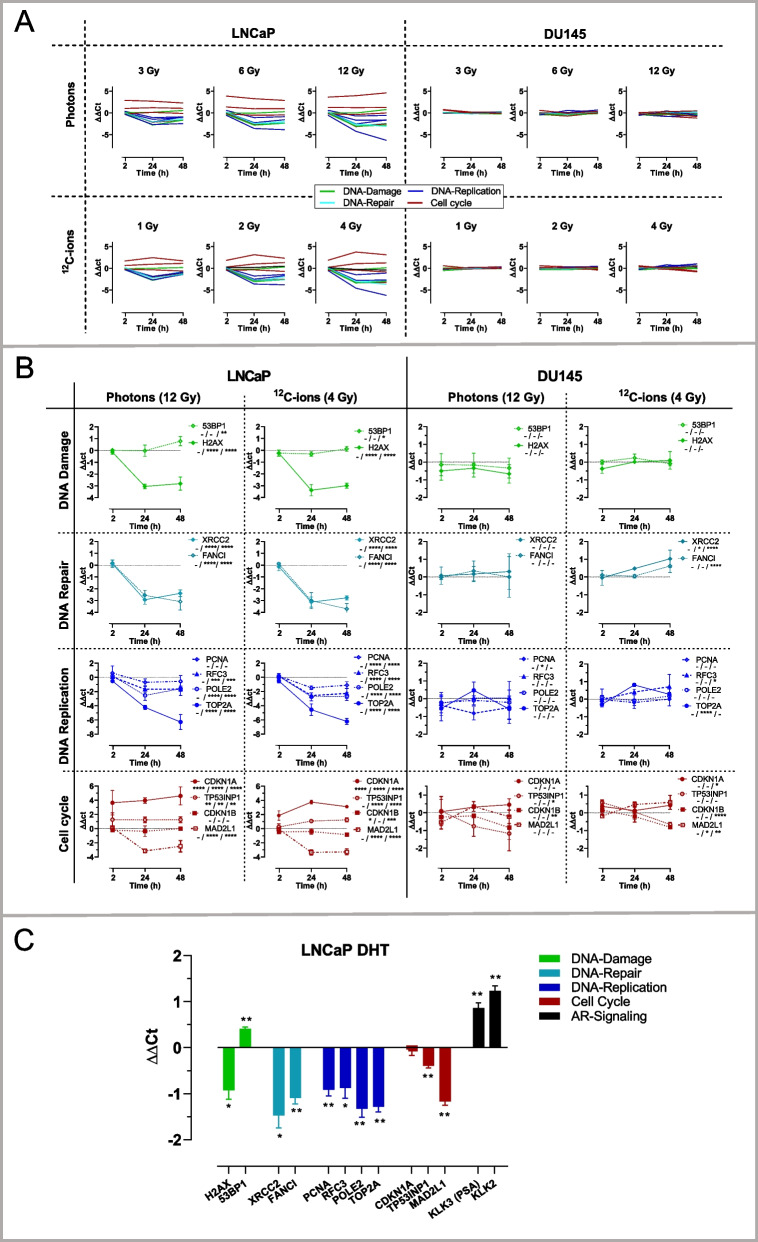
RT-qPCR analysis of selected mRNAs related to DNA-damage (green), DNA-repair (cyan), DNA replication (blue) and cell cycle (red) in LNCaP (left) and DU145 cells (right). **A** Overview of the changes of mRNA levels (ΔΔCt, mean, n = 3) upon photon and ^12^C-ion irradiations with all tested doses and time points. **B** Subsets of changes of mRNA levels (ΔΔCt) (mean ± SD, n = 3) in adjusted scales displayed for the irradiation modes with the maximum applied dose for photons (12 Gy) and for ^12^C-ions (4 Gy). Significant differences (2-way-ANOVA with multiple comparisons) are indicated for each time point (separated by slash) in the insets. **C** Changes of mRNA levels (ΔΔCt) after DHT treatment (20 nM; 24 h) of LNCaP cells for analysis of mRNAs related to DNA-damage (green), DNA-repair (cyan), DNA replication (blue), cell cycle (red) in addition to androgen receptor (AR) signaling (black). The changes of mRNA levels (ΔΔCt) are displayed (mean ± SD, n = 3). Significant differences between DHT and control were determined by unpaired Welch corrected t-test. Symbols of significances (*p < 0.05, **p < 0.01; ***p < 0.001; ****p < 0.0001)

Next, we analyzed selected mRNAs of DNA-damage, DNA-repair, DNA replication and cell cycle in LNCaP and DU145 cells. An overview of the changes in mRNA levels upon photon and ^12^C-ion irradiation with all tested doses and time points is displayed (Fig. 3A). It reveals that the mRNA changes in LNCaP cells are strikingly stronger than in DU145 cells.

We further divided the respective subsets of mRNAs and displayed their changes in adjusted scales for LNCaP and DU145 cells (Fig. 3B). Interestingly, in LNCaP cells, the initial increase in H2AX protein that was observed after 2 h (Fig. 3C) was counterbalanced by decreased H2AX-mRNA levels at later time points (24 h, 48 h) (Fig. 3B, 1*st lane, left*) whereas the 53BP1-mRNA levels were slightly upregulated (< twofold). In DU145 cells, both H2AX-mRNA and 53BP1-mRNA levels were unaffected by irradiation (Fig. 3B, 1*st lane*, right).

In addition to H2AX and 53BP1, we investigated additional mRNAs by RT-qPCR that are involved in DNA repair (XRCC2, FANCI) (Fig. 3B; *2nd lane*), DNA replication (PCNA, RFC3, POLE2, TOP2A) (Fig. 3B; *3rd lane*) and cell cycle control (CDKN1A, CDKN1B, TP53NIP1, MAD2L1) (Fig. 3B, 4*th lane*).

**Fig. 4:**
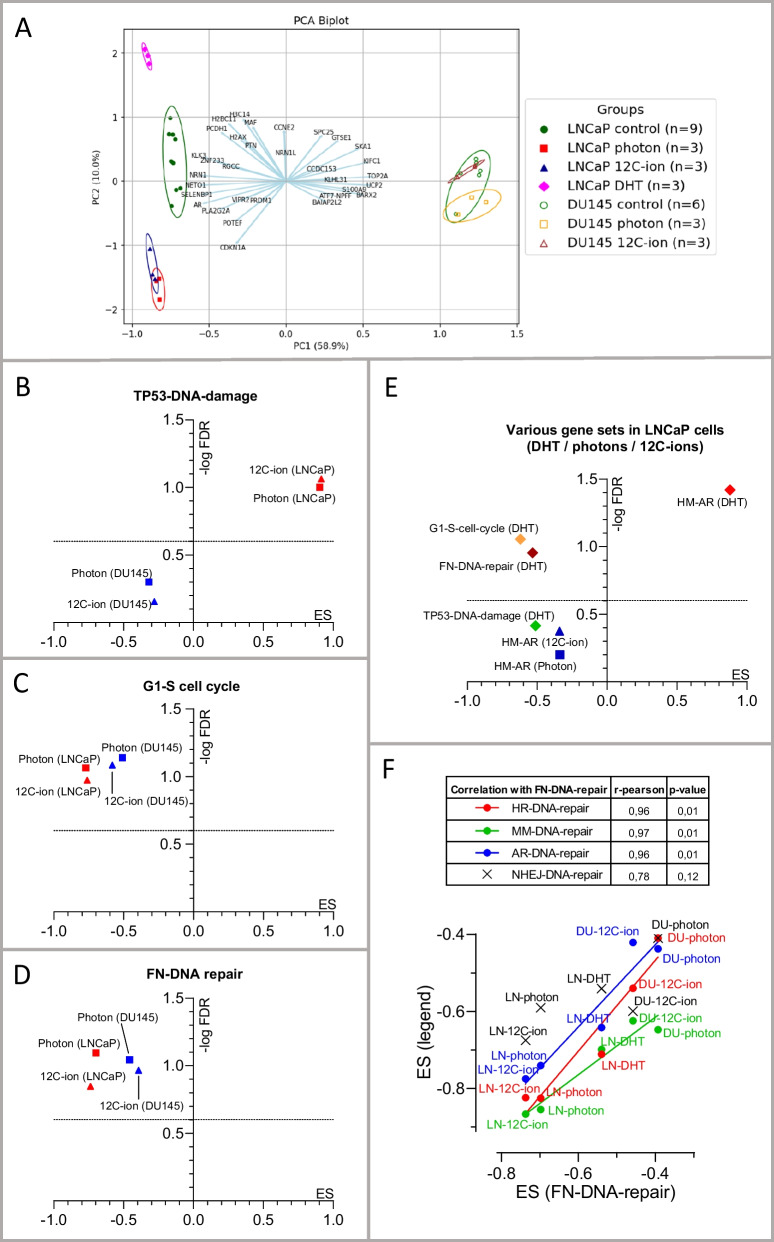
Analysis of RNA seq data by Principal Component Analysis (PCA) and gene set enrichment analysis (GSEA) from samples of control, photon (6 Gy; 24 h) and ^12^C-ion (2 Gy; 24 h) irradiation or DHT treatment (20 nM; 24 h). **A** PCA Biplot of the mRNAs including all samples. The *principal component 1* (PC1) covers 58.9% (x-axes) and PC2 covers 10% (y-axes) of the multidimensional data sets of the detected mRNA targets (n > 10^4^). The samples of LNCaP or DU145 cells are grouped as control, photons, ^12^C-ions or DHT, as indicated by different colors and symbols (see legend). The n-number of controls is n = 9 for LNCaP and n = 6 DU145 since the respective matched control values (n = 3) were merged. The samples are encircled by a line representing the 95% confidence interval (95% CI) for each group. The loading vectors (light blue) represent several selected altered genes and indicate their contribution to PC1 or PC2. (B-E) Gene set enrichment analyses (GSEA) of enrichment score (ES) and false discovery rate (FDR) are displayed as volcano plots (ES vs. − log FDR). In GSEA, ES-values are considered as significant if FDR < 0.25 and *p* < 0.05. The dotted line at (− log FDR = 0.602) corresponds to FDR = 0.25, meaning that the ES values above this line have an FDR < 0.25. Here, these gene sets have nominal *p*-values < 0.01 (see supplementary information) and therefore the ES-values above the dotted line represent significant ES-values. The critical ES-values of the treatments are displayed with respect to different gene sets: **B** *Amundson-DNA-damage-response-TP53* (TP53-DNA-damage) for photons and ^12^C-ions; **C** *WP-G1-to S-cell cycle-control* (G1-S-Cell cycle) photons and ^12^C-ions; **D** *WP-DNA-repair pathway full network* (FN-DNA-Repair) for photons and ^12^C-ions. In **E** the *Hallmark Androgen Response* (HM-AR) in DHT-treated LNCaP cells is compared with photon and ^12^C-ion irradiated LNCaP cells. In **E**, the gene sets shown in **B**–**D** are analyzed with respect to DHT treatment. In **F**, the ES values of FN-DNA-repair are correlated with those of other DNA-repair modes particularly, *DNA-repair-homologue-recombination* (HR-DNA-repair), *non-homologue-end-joining* (NHEJ-DNA-repair), *mismatch repair-DNA-repair* (MM-DNA-repair) and *AR-targeted DNA-repair* (AR-DNA-repair). The symbols refer to the DNA-repair mode (see inset table). Treatments and cells are indicated LNCaP (LN) and DU145 (DU)

In LNCaP cells (Fig. 3B, *left side*), the mRNAs of DNA-repair- (XRCC2, FANCI) and DNA-regulator-genes (TOP2A, RFC3, POLE2; MAD2L1) except for PCNA were downregulated by both ^12^C-ion and photon irradiation. In particular, TOP2A mRNA was downregulated by almost 100-fold (ΔΔCt > 6). As a critical cell cycle inhibitor, CDKN1A-mRNA was upregulated by approximately 20-fold (ΔΔCt > 4) upon photon and ion irradiation.

In DU145 cells (Fig. 3B, *right side*), the alterations of mRNAs after irradiation with ^12^C-ions and photons were of considerably lower amplitude (< twofold; ΔΔCt < 1) than in LNCaP cells and are adequately displayed in a narrower scale.

### Regulation of selected mRNAs by DHT

For evaluating AR-signaling, LNCaP cells were treated with DHT (Fig. 3C). The AR markers KLK3 (prostate specific antigen, PSA) and KLK2 were measured as known AR targets and displayed the typical DHT-dependent induction. In the DNA repair group, mRNAs for XRCC2, FANC1 and H2AX were significantly downregulated (twofold), while 53BP1 was slightly upregulated (1.5-fold) by DHT. Similarly, the DNA regulators PCNA, RFC3, POLE2 and TOP2A were downregulated (twofold). In the cell cycle group, CDKN1A was unaffected, whereas T53INP and MAD2L1 were downregulated by DHT.

In essence, certain genes from functional groups gave typical congruent responses in LNCaP cells to ^12^C-ion and photon irradiation. In particular, some DHT responses were common to the irradiation responses, most distinctly for DNA-damage and DNA repair genes and partially for DNA-regulators and cell cycle genes (e.g. TOP2A, MAD2L), with the exception of CDKN1A. In contrast to LNCaP cells, DU145 cells showed considerably lower changes in mRNA levels following irradiation.

### Transcriptomic analysis by RNA-seq

Subsequently, we analyzed the transcriptomic mRNA response of LNCaP and DU145 cells by RNA-seq. Based on the previous results (Fig. 3A, B), the analysis was performed 24 h after irradiation with a dose of 6 Gy (photons) and 2 Gy (^12^C-ions). These intermediate irradiation doses as well as the intermediate time period exhibited significant gene regulatory effects and appeared suitable for comprehensive RNA-seq analysis. In particular, the higher selected dose range of photons versus ^12^C-ions is supported by literature [23, 27]. For analysis of AR response, LNCaP cells were incubated with 20 nM DHT. Each treatment included directly matched naïve control cells for analysis.

The obtained RNA seq data were evaluated using *Principal Component Analysis* (PCA) (Fig. 4A). PCA projected the huge multidimensional mRNA data (n > 10^4^) of the treated and control samples to a 2-dimensional plane geometry with maximum possible differentiation for the first dimension 1 (X-axis: PC1 = 58.9%) and for the second dimension (Y-axis: PC2 = 10%) (Fig. 4A). The samples (dots) were labeled according to cell type and treatment group (control, photons, ^12^C-ions, or DHT). The groups of samples are encircled by respective lines indicating the 95% confidence interval (CI) of the treatment. In result, LNCaP cells (allocated on the left side) and DU145 cells (allocated on the right side) were separated. The LNCaP cells (filled shapes) could be separated into (i) control group (green circles) (ii) DHT group (red diamonds) and (iii) the overlapping irradiation modes ^12^C-ion (blue triangles) and photon (purple squares). Within DU145 cells (empty shapes), no separation was possible since the encircled 95% CI of control (green circles), photon (yellow squares) and ^12^C-ion (brown triangles) all overlapped. The loading vectors (light blue) refer to the most differentially expressed genes. Some of these have been already mentioned in Fig. 3 (CDKN1A, KLK3, TOP2A).

### Gene set enrichment analysis of RNA-seq data

Furthermore, the RNA-seq data were analyzed using Gene set enrichment analysis (GSEA). GSEA statistically analyses *gene sets* that are preferentially altered as a whole in response to a trigger. Here, we focused on gene sets covering signaling pathways that are related to irradiation and AR signaling (listed in supplementary information). We tested gene sets related to *Amundson-DNA-damage-response-TP53* (TP53-DNA-damage), *WP-DNA-repair-pathway-full-network* (FN-DNA-repair), *WP-G1-to S-cell cycle-control* (G1-S-cell cycle), and *hallmark-androgen-response* (HM-AR). Individual members of these pathways have been addressed previously (Fig. 3).

### TP53-DNA-damage gene set

LNCaP cells displayed a significant positive enrichment score for the gene set TP53-DNA-damage after photon and ^12^C-ion-irradiation (Fig. 4B). In DU145 cells, which are dysfunctional for TP53, no significant ES for TP53-DNA-damage by photons or ^12^C-ions was determined (Fig. 4B).

### G1-S-Cell-cycle gene set

LNCaP cells displayed significant negative ES for the gene set G1-S-cell cycle by photons and ^12^C-ions (Fig. 4C). In DU145 cells, this gene set also displayed significant negative ES, but to a lesser extent, when treated with photons and ^12^C-ions (Fig. 4C).

### DNA-repair gene set

Next, we analyzed the gene set FN-DNA-repair. LNCaP cells showed significant negative ES after photon and ^12^C-ion-irradiation (Fig. 4D). In DU145 cells, the gene set FN-DNA-repair also displayed significant negative ES for both photons and ^12^C-ions (Fig. 4D).

### *DHT, photons and *^*12*^*C-ions in relation to several gene set pathways*

The androgen responsive LNCaP cells that had been treated with DHT were analyzed with respect to androgen receptor signaling using the gene set HM-AR (Fig. 4E). As expected, the gene set HM-AR exhibited a significant positive ES in DHT treated LNCaP. In comparison, the gene set HM-AR showed rather negative ES for photons and ^12^C-ions without significance. Alongside, TP53-DNA-damage, FN-DNA-repair and G1-S-cell cycle were analyzed for DHT treated LNCaP cells (Fig. 4E). TP53-DNA-damage was not significantly altered by DHT, whereas the ES values for FN-DNA-repair and G1-S-cell cycle control revealed significant negative enrichment by DHT. Complementarily, photons and ^12^C-ions both had negative ES-values for HM-AR but without significance (Fig. 4E). Furthermore, we compared FN-DNA-repair with subsets of DNA-repair modes including gene sets for *kegg-homologues-recombination* (HR-DNA-repair), *kegg-non-homologues-end-joining* (NHEJ-DNA-repair), *kegg-mismatch-repair* (MM-DNA-repair) and the subset of *AR-targeted DNA-repair* genes (AR-DNA-repair) [9]. The ES-values of FN-DNA-Repair significantly correlated with the listed DNA-repair modes except for NHEJ-DNA-repair (Fig. 4F).

### *Differentially expressed genes (DEG) after irradiation by photons or *^*12*^*C-ions and DHT treatment*

**Fig. 5:**
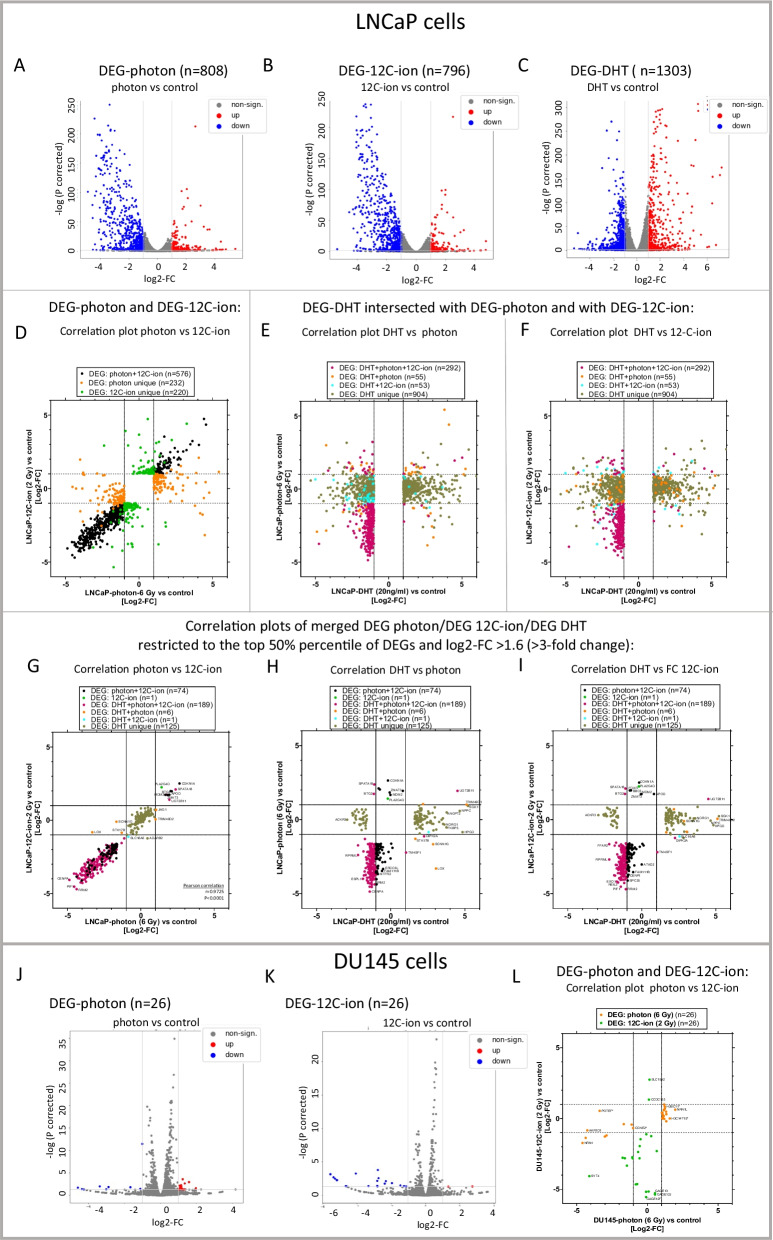
Analysis of differentially expressed genes (DEG) determined by RNA-seq. DEG of LNCaP cells for DEG-photon **A**, DEG-^12^C-ion **B** and DEG-DHT **C** and in DU145 cells for DEG-photon **J** and DEG-^12^C-ion **K**. DEG are defined as log2FC ≥ 1; corrected *p* < 0.05. The log2-FC in the heatmaps with the corresponding volcano plots indicate treatment versus control (n = 3). Upregulated DEG are red, downregulated DEG are blue and non-significantly altered mRNAs are grey. **D** Correlation graphs of log2-FC of DEG-photon and DEG-^12^C-ion in LNCaP cells. DEG-photon that intersected with DEG-^12^C-ion are black (n = 577). Unique DEG-photon are orange (n = 232) and unique DEG-^12^C-ion are green (n = 220). **E**, **F** Correlation graphs of DEG-DHT that intersected with DEG-photon or with DEG-^12^C-ion. The same samples are plotted once with (log2-FC-photon) on the y-axis **E** and once with (log2-FC-(^12^C-ion) on the y-axis **F**. The majority of DEG-DHT were unique (n = 904, olive). A prominent fraction of DEG-DHT intersected with DEG-^12^C-photon (orange; n = 55), with DEG ^12^C-ion (cyan; n = 53) or with both (red; n = 292). **G**–**I** Correlation graphs of merged DEG-photon, DEG-^12^C-ion and DEG-DHT that are restricted to those with high expression levels (top 50th percentile of all DEG) and strong fold changes (0.3 > FC > 3; adequately to (log2FC > 1.6) for at least one treatment. The samples are plotted with different XY-axes. **G** log2-FC of photon versus ^12^C-ion with Pearson correlation analysis (r = 0.9725, *p* < 0.0001). **H** log2-FC of DHT versus photon and **I** log2-FC of DHT versus ^12^C-ion. Respective unique and intersected DEG are assigned to different sample colors with the n-number in the legends. The names of strikingly altered DEG are indicated. **L** Correlation graphs of log2-FC of DEG-photon and DEG-^12^C-ion in DU145 cells. DEG-^12^C-ion (orange; n = 26) and DEG-^12^C-ion (green, n = 26) were all unique without intersection. Some DEG-photon in DU145 cells (n = 5) intersected with DEG-photon in LNCaP cells and are marked with an asterisk

Next, *DEG* were determined from the RNA-seq data. DEG are defined as mRNAs that are altered with a log2FC ≥ 1 and corrected *p*-values < 0.05. DEG-photon and DEG-^12^C-ion were determined for LNCaP (Fig. 5A, B) and for DU145 cells (Fig. 5J, K). Furthermore, DEG-DHT were determined for LNCaP cells (Fig. 5C). The DEG are displayed in volcano plots.

### DEG in LNCaP cells

Essentially, LNCaP cells (with functional TP53 and AR signaling) displayed considerable alterations of certain mRNA levels upon photon and ^12^C-ion irradiation. A higher proportion of DEG-photon (n = 809) and of DEG-^12^C-ion (n = 797) were downregulated (blue) than upregulated (red) (Fig. 5A, B). Concerning DEG-DHT (n = 1304), a higher proportion was upregulated (red) than downregulated (blue) (Fig. 5C). The individual names of DEG are listed (supplementary information).

Furthermore, correlation plots depicting the fold-change (FC) of DEG-photon, DEG-^12^C-ion and DEG-DHT were constructed. DEG-photon considerably overlapped with DEG-^12^C-ion (black; n = 577) (Fig. 5D). DEG-photon that were unique are labeled in orange (n = 232) and those that were unique for DEG-^12^C-ion are labeled in green (n = 220) (Fig. 5D). Moreover, we compared DEG-DHT with DEG-photon or DEG-^12^C-ion; once with log2-FC-photon on the Y-axis (Fig. 5E) and once with Log2-FC-^12^C-ion on the Y-axis (Fig. 5F). We found that the majority of DEG-DHT were unique (n = 904, olive). A prominent fraction of DEG-DHT intersected with DEG-^12^C-photon (orange; n = 55), with DEG ^12^C-ion (cyan; n = 53) or with both (purple; n = 292).

Next, we focused on those DEG that have high expression levels and are strongly changed upon treatments. To that end, we arbitrarily restricted the comparison to the DEG of the top 50th percentile with a minimum threefold change *(log2FC* > *1.6)* under photon, ^12^C-ion or DHT treatment. These DEGs were then merged and displayed in three XY-graphs (Fig. 5G, H, I). Strikingly, a strong correlation between DEG-photon versus DEG-^12^C-ion became obvious in LNCaP cells (Pearson r = 0.9725; *p* < 0.0001) reflecting the particularly high degree of congruent regulation by ^12^C-ion and photon in the top level DEG. The extremely altered mRNAs are named (Fig. 5G). Alongside, we displayed the values of the same samples for log2-FC DHT (X-axes) versus log2-FC photon (Y-axes) (Fig. 5H) and for log2-FC DHT (X-axes) versus log2-FC ^12^C-ion (Y-axes) (Fig. 5I). It is noteworthy that the genes that are predominantly induced by DHT remain unaffected by photon- or ^12^C-ion-irradiation (e.g.: SGK1, NPPC, HPGD, NDRG1). On the other hand, those genes that are majorly induced by photons and ^12^C-ions remain unaffected by DHT (e.g. CDKN1A, ZMAT3, MDM2) (Fig. 5H, I).

### DEG in DU145 cells

In DU145 cells (dysfunctional TP53), the number of DEG-photon (n = 26) and DEG-^12^C-ions (n = 26) (Fig. 5J, K) were significantly lower (1–2 magnitudes) than in LNCaP cells (Fig. 5A, B). In addition, the amplitude of the fold changes was considerably lower and the triplicate values of fold changes appear less conform. Furthermore, all DEG-photon and DEG-^12^C-ion were unique in DU145 cells without intersection. The DEG showing the most extreme fold changes are identified with labels. Some mRNAs (n = 5) of DEG-photon in DU145 cells intersected with DEG-photon in LNCaP cells and are indicated by an asterisk in the correlation graph (Fig. 5L). For DEG-^12^C-ion, no intersection was observed between DU145 and LNCaP cells.

### *Identification of putative *^*12*^*C-ion and photon dependent pathways in LNCaP and DU145 cells*

As a direct approach to identify putative ^12^C-ion and photon dependent pathways, GSEA was applied to ^12^C-ion irradiated versus photon irradiated samples. This allowed us to identify *Notch signaling* as significantly enriched in ^12^C-ion versus photon irradiated LNCaP cells. In DU145 cells, we identified the *unfolded protein response*, the r*eactive oxygen species pathway* and o*xidative phosphorylation* as significantly enriched in ^12^C-ion versus photon irradiated samples (supplementary information).

## Discussion

Photon irradiation is an established technique for the treatment of prostate cancer and ^12^C-ion irradiation represents an innovative radiation technique. The physical parameters of these irradiation modes are well defined and their performance in radiotherapy of patients with advantages and disadvantages have been documented [3, 4].

Here, we rather focussed on critical biological responses in prostate cancer cells. As an early response, we compared DNA-damage of the applied irradiation doses of photons versus ^12^C-ions. As a later manifesting response, we studied gene regulation related to DNA-damage, DNA-repair, cell cycle and AR-signaling that is relevant in prostate cancer progression. The key messages are briefly listed and subsequently discussed.

i. The applied doses of photons and ^12^C-ions were suitable in terms of inducing adequate DNA damage and gene responses. Alongside, these conditions caused typical induction of TP53 DNA-damage pathway by both photons and ^12^C-ions in TP53 functional cells, but not in TP53 dysfunctional cells.
ii. The gene sets of DNA-repair and G1-S-cell-cycle were downregulated by photon- and ^12^C-ion-irradiation in both TP53-functional and TP53-dysfunctional cells.
iii. AR-signaling was typically induced by DHT and this was paralleled by downregulation of the gene sets of DNA-repair and G1-S-cell-cycle. Photons and ^12^C-ions did not cause significant changes in canonical AR-signaling gene sets.
iv. The identified uniquely regulated genes by photons and ^12^C-ions may serve as biological markers for the physical differences between photon and ^12^C-ion irradiation. In addition, GSEA-based analysis directly comparing ^12^C-ion and photon irradiation suggests some differentially regulated signaling pathways.

### *Dose ranges of photons and *^*12*^*C-ions in relation to biological responses*

The selected dose ranges of photons (3–6–12 Gy) and ^12^C-ions (1–2–4 Gy) caused comparable amounts of DSB-foci in LNCaP and DU145 cells, as well as an increased level of cellular H2AX protein within the cells which is in-line with an observation made for photons [28].

With respect to gene regulation of LNCaP cells, the selected mRNAs displayed a plateau of changes in the applied dose ranges for photons (6–12 Gy) and ^12^C-ions (2–4 Gy). In addition, the time frame (2–24–48 h) covered the critical range due to its asymptotic dynamics (Fig. 3A, B). Additionally, correlation analysis of RNA-seq data confirmed almost equal changes of the intersected mRNAs (DEG) upon exposure to photons and ^12^C-ions in LNCaP cells (r = 0.9725, *p* < 0.0001 in Fig. 5G). Thus, the dose ranges align with the data for RBE demonstrated to be lower for photons than for ^12^C-ions [23, 27]. Apparently, (i) the early response to irradiation indicated by DSB, (ii) the later response manifested at the level of gene regulation and (iii) the final response manifested by cell death (RBE) are roughly related.

### DNA damage and TP53 status

The greater amount of DNA damage observed in LNCaP compared to DU145 cells following exposure to photons and ^12^C-ions is consistent with previous findings for photons [29]. Speculatively, DNA repair may be initiated more rapidly in DU145 cells than in LNCaP cells, so that the repair process is already evident at the first recorded time period of 2 h post-irradiation [30]. Another explanation may be related to the TP53 status [31]. Naturally, the TP53-DNA damage signaling was strikingly responsive towards photons and ^12^C-ions in LNCaP (functional TP53), but not in DU145 cells (dysfunctional TP53) (Fig. 3B, Fig. 4B). Of relevance, TP53 was suggested to break up DNA condensation by disassembling protective tight protein/DNA interactions, resulting in higher resistance to DNA-damage in TP53 dysfunctional cells [31, 32].

### *DNA damage upon photon and *^*12*^*C-ion irradiation and DNA-repair mechanisms*

Several studies suggested that DNA-damage caused by ^12^C-ions and photons differ with respect to the specific mechanisms of DNA-repair such as HR-DNA-repair and NHEJ-DNA repair. However, these studies are not consistent [23, 33–35]. Generally, our study revealed negative enrichment scores for various gene subsets of DNA-repair upon photons and ^12^C-ions with differences summarized in the correlation analysis (Fig. 4F). As a limitation of our study, the connection between the regulation of certain DNA-repair genes and functional DNA-repair remains elusive and would require biochemical data of the active DNA-repair mechanisms for explanation. The observed downregulation of DNA-repair genes upon photon irradiation in prostate cancer cells has been described elsewhere [36]. As a potential mechanism for downregulation in a distinct context, it was demonstrated that irradiation induced miR-711, which subsequently inhibited several DNA-repair genes [37]. Based on our data, we hypothesize that the regulation of specific targets for DNA repair and damage may diverge between protein and mRNA as exemplified for the increased expression of H2AX protein alongside the decreased level of H2AX-mRNA (Figs. 2B, C). The diverging levels of the related protein and mRNA markers may reflect active regulatory adaptation to the disturbed condition.

### *Regulation of AR-signaling by DHT in relation to photons and *^*12*^*C-ions*

The HM-AR gene set pathway was not significantly affected by photons or ^12^C-ions in LNCaP cells. The DHT-treated LNCaP group served as a control for HM-AR gene set induction. Similarly, the DNA-damage-TP53 pathway was not significantly affected by DHT. However, analogous to the irradiations, DHT did suppress DNA-repair and G1-S cell cycle gene sets. Specifically, the subsets of the DNA-repair pathways AR-DNA-repair, HR and MMR were significantly suppressed by DHT. Upon initial review, our findings differ from a previously conducted study that demonstrated AR-dependent induction of DNA-repair gene sets [9]. The aforementioned study utilized the AR agonist R1881 at a concentration of 1 nM, which resulted in the induction of the AR-DNA-repair gene set [9]. Methodologically different, we used the cellular agonist DHT at supraphysiological dose (20 nM) and importantly the AR-DNA-repair gene set in addition to other DNA-repair gene sets were significantly downregulated by DHT. Our results are quite robust and the experimental performance was corroborated by the typical induction of the AR pathway through DHT (Fig. 4E). A related study showed that employing supra-physiological doses of DHT (up to 100 nM) caused DNA-damage [10]. Another study demonstrated that exposure of LNCaP cells to high dose of R1881 (100 nM) blocked LNCaP proliferation, an effect that could be attributed to cell cycle dependent proteasomal degradation of AR [38, 39]. These fundamental observations may be associated with the results of a clinical trial [40, 41] where bipolar androgen therapy with alternating supraphysiological R1881 doses versus castrate-serum androgen levels revealed a benefit for patients with prostate cancer. Overall, our data indicate that using supra-physiological doses of DHT, as opposed to the lower androgen doses found in literature [9], results in the downregulation of DNA-repair genes. In this regard, DHT has similar effects on DNA repair genes as photon and ^12^C-ion irradiation.

### *Limitations of the study with respect to *^*12*^*C-ion and photon dependent signaling pathways*

The limitations of the study relate to the general meaning of differentially expressed genes reflecting responses towards photon and ^12^C-ion irradiation in only two genetically different prostate cancer cell lines. An interesting finding is that these two cell lines considerably differ in their transcriptomic response towards the irradiation modes between each other.

Furthermore, GSEA-based analysis was used to directly compare ^12^C-ion and photon irradiation and this approach identified differences in the magnitude of certain gene responses (supplementary information). In particular, responses related to N*otch signaling,* u*nfolded protein response*, r*eactive-oxygen-species pathway* and o*xidative phosphorylation* differed significantly between the irradiation modes in a cell type-specific manner. *Notch signaling* is altered in several types of cancer and promotes epithelial-mesenchymal transition and angiogenesis [42]. The *unfolded protein response* of the endoplasmic reticulum maintains a healthy proteome and its disturbance is relevant in the pathogenesis of cancer [43]. The *reactive oxygen species pathway* is frequently disrupted in cancer cells and is affected by irradiation [44]. *Oxidative phosphorylation* along with glycolysis are altered in cancer cells as well [45]. The cause and exact biological significance of the differently altered signaling pathways between the irradiation modes is currently still unknown. It is noteworthy that the observed differential regulation of these pathways in direct comparison of the irradiation modes would need to be examined in more detail in a dose-dependent manner for confirmation.

## Conclusion

In conclusion, this study demonstrated similar gene regulatory responses to photon and ^12^C-ion irradiation with respect to DNA-damage, DNA-repair and cell cycle, with specificity to the utilized cell type. Androgen receptor-signaling and irradiations shared basic responses with respect to gene sets of DNA-repair and G1-S-cell-cycle. Alongside, distinctive mRNA alterations elicited through photons versus ^12^C-ions are suggested.

### Supplementary Information

Additional file 1.

## Data Availability

All the data supporting the findings of this study are available within the paper. The raw data of RNA-seq has been submitted to EMBL Biostudies and can be accessed via the accession number E-MTAB-14099.

## Abbreviations

AR: Androgen receptor
53BP1: DSB marker protein
^12^C-ions: Carbon ions
CDKN1A: Cell cycle kinase inhibitor
CI: Confidence interval
DEG: Differentially expressed gene
DHT: (5α, 17β)-17-Hydroxy-androstane-3-one (di-hydro-testosterone)
DSB: DNA double strand break
ES: Enrichment score
FC: Fold change
FDR: False discovery rate
GSEA: Gene set enrichment analysis
H2AX: DSB marker protein
PCA: Principal component analysis
